# Shear Viscosity of Uniform Fermi Gases with Population Imbalance

**DOI:** 10.1038/s41598-018-22273-1

**Published:** 2018-03-05

**Authors:** Weimin Cai, Hao Guo, Yan He, Chih-Chun Chien

**Affiliations:** 1 Department of Physics, Southeast University, Nanjing, 211189 China; 2 College of Physical Science and Technology, Sichuan University, Chengdu, Sichuan 610064 China; 3 School of Natural Sciences, University of California, Merced, CA 95343 USA

## Abstract

The shear viscosity has been an important topic in ultracold Fermi gases, and it has served as a diagnostic of various theories. Due to the complicated phase structures of population-imbalanced (polarized) Fermi gases with tunable attraction, past works on the shear viscosity mainly focused on unpolarized Fermi gases. Here we investigate the shear viscosity of homogeneous, population-imbalanced Fermi superfluid at finite temperatures by a pairing fluctuation theory for thermodynamical quantities and a gauge-invariant linear response theory for transport coefficients. The Cooper pairs lead to the anomalous shear viscosity analogous to the shear viscosity. We derive an exact relation connecting certain thermodynamic quantities and transport coefficients at the mean-field level for polarized unitary Fermi superfluids. An approximate relation beyond mean-field is proposed and only exhibits mild deviations from our numerical results. In the unitary and Bose-Einstein condensation (BEC) regimes, the total shear viscosity increases with the polarization because the excess majority fermions cause gapless excitations acting like a normal fluid. Moreover, competition among the excess fermions, noncondensed pairs, and fermionic quasiparticles may lead to non-monotonic behavior of the ratio between the shear viscosity and relaxation time as the polarization increases.

## Introduction

Ultracold atomic Fermi gases provide versatile quantum simulators for complex many-particle systems, and their transport properties have attracted broad research interest^1–19^. The shear viscosity relating the momentum transfer transverse to a shear force is an important subject, and it helps test many-body theories. Moreover, the ratio of the shear viscosity to entropy density of unitary Fermi gases when the fermions are about to form two-body bound states has been shown to be close to a quantum lower bound^1,6,20^. Previous theoretical works, however, mainly focus on the shear viscosity of two-component Fermi gases with equal populations^8,16,21–25^. Since the populations of different components of ultracold atomic Fermi gases can be adjusted^26–31^, population-imbalanced Fermi gases have been an intensely studied subject in cold-atoms. The studies of population-imbalanced Fermi gases are more difficult since at low temperatures phase separation of paired and unpaired fermions can emerge^28,30^. The “intermediate-temperature superfluid” describes where homogeneous polarized superfluids appear^32^ when a population-imbalanced ultracold Fermi gas undergoes the BCS-Bose Einstein condensation (BEC) crossover as the attractive interaction increases^33^. Here, we focus on 3D Fermi gases and mention that other exotic phases and structures may emerge in 1D population-imbalanced Fermi gases^34^.

A theoretical study of the shear viscosity of ultracold atomic Fermi superfluid with population imbalance will be presented here. We integrate the thermodynamics from a pairing theory of a fermionic superfluid beyond the mean-field BCS theory and the transport coefficients from a gauge-invariant linear response theory called the consistent fluctuation of order parameter (CFOP) theory^35,36^. In the unitary and BEC regimes, preformed (noncondensed) pairs emerge at finite temperatures due to strongly attractive interactions, but they do not contribute to superfluidity. Theories incorporating the noncondensed pairs are often called pairing-fluctuation theories^37–39^. The noncondensed pairs can lead to an energy gap in the single-particle dispersion, usually called the pseudogap^40^, even in the absence of superfluidity. Thus, the pairing energy gap Δ should be distinguished from the order parameter Δ_sc_ describing the condensed coherent Cooper pairs. As a consequence, the pairing onset temperature T* is higher than the superfluid transition temperature *T*_*c*_ in the strongly attractive regime. Among various approaches, one pairing-fluctuation theory consistent with the Leggett-BCS ground state^33,41^ has been successfully applied to polarized Fermi gases in the BCS-BEC crossover^32,42^; we will implement it here.

The shear viscosity should include both the fermionic and bosonic (from the noncondensed pairs) contributions in the strongly attractive regime^43^. In addition, the Cooper pairs result in the anomalous shear viscosity similar to the shear viscosity from the energy-momentum tensor response function^43^. (See also the Supplemental Information.) The total shear viscosity of polarized Fermi superfluids should include all the contributions, and we will show how a competition between the excess majority fermions and various excitations affect the ratio between the viscosity and relaxation time.

Recently, a relations connecting the shear viscosity *η* and normal-fluid density *n*_*n*_ for unpolarized Fermi gases, *η = n*_*n*_*D* with *D* denoting the diffusion constant, has been discussed in refs^24,44^. To help constrain the relaxation time which appears in linear response theory, we generalize another relation^43^ connecting certain thermodynamic quantities, transport coefficients, and relaxation time to polarized unitary Fermi superfluids. An exact relation at the mean-field level will be presented, but the beyond mean-field case is quite complicated. We instead present an approximate relation including the contributions from the noncondensed pairs.

## Results

### Thermodynamics of polarized Fermi gases

At the mean-field (BCS) level, the equations of state of an attractive two-component (labeled by *σ* = ↑,↓) population-imbalanced Fermi gas include two number equations and a gap equation^5,45–47^. Assuming the two components have the same fermion mass *m* and densities *n*_↑,↓_, the equations are given by (*n* ≡ *n*_↑_ + *n*_↓_ and δ*n* ≡ *n*_↑_ − *n*_↓_)

1 $$n=\sum _{{\bf{k}}}\,[1-\frac{{\xi }_{{\bf{k}}}}{{E}_{{\bf{k}}}}\mathrm{(1}-2\bar{f}({E}_{{\bf{k}}}))],\,\delta n=\sum _{{\bf{k}}}(f({E}_{{\bf{k}}\uparrow })-f({E}_{{\bf{k}}\downarrow })),\,\frac{1}{g}=\sum _{{\bf{k}}}\frac{1}{2{\varepsilon }_{{\bf{k}}}}-\frac{m}{4\pi a}=\sum _{{\bf{k}}}\frac{1-2\bar{f}({E}_{{\bf{k}}})}{2{E}_{{\bf{k}}}},$$

where $\mu =\frac{{\mu }_{\uparrow }+{\mu }_{\downarrow }}{2}$, $h=\frac{{\mu }_{\uparrow }-{\mu }_{\downarrow }}{2}$, *ε*_***k***_ = *k*^2^/2*m*, *ξ*_**k**_ = *ε*_**k**_ − *μ*, ${E}_{{\bf{k}}}=\sqrt{{\xi }_{{\bf{k}}}^{2}+{{\rm{\Delta }}}^{2}}$, ${E}_{{\bf{k}}\uparrow ,\downarrow }={E}_{{\bf{k}}}\,\mp \,h$, *f*(*x*) = 1/(1 + *e*^*x*/*T*^) is the Fermi distribution function, and $\bar{f}(x)=(f(x+h)+f(x-h\mathrm{))/2}$. There is no distinction between the order parameter and energy gap at the mean-field level, so we use Δ to denote both. Here *ћ* = 1, *k*_*B*_ = 1, *K* = (*iω*_*n*_, **k**), *Q* = (*i*Ω_*l*_, **q**) and ${\sum }_{K}=T{\sum }_{{\omega }_{n}}\,{\sum }_{{\bf{k}}}$, where *ω*_*n*_ (Ω_*l*_) is the fermionic (bosonic) Matsubara frequency. Moreover, *μ*_*σ*_ is the chemical potential for each component (spin), *g* is the attractive coupling constant modeling the contact interaction between atoms, and *a* is the two-body *s*-wave scattering length. The unitary limit is determined by 1/(*k*_*F*_*a*) = 0, where *k*_*F*_ is the Fermi momentum of a noninteracting Fermi gas with the same density and ${E}_{F}={\hslash }^{2}{k}_{F}^{2}\mathrm{/(2}m)$ is its Fermi energy. The BCS (BEC) regime corresponds to 1/(*k*_*F*_*a*) < 0 (1/(*k*_*F*_*a*) > 0). In addition, the ground state of a polarized Fermi gas can exhibit phase separation of paired and unpaired fermions in the BCS and unitary regimes^5,42,48^.

There are challenges and opportunities for theories of strongly attractive Fermi gases beyond the mean field approximation. A t-matrix theory known as the Nozieres Schmitt-Rink (NSR) theory has been applied to polarized Fermi gases in the BCS-BEC crossover^47^. However, negative compressibility was produced from the NSR theory around the unitary point. A systematic treatment using the large-N expansion has been applied to unpolarized Fermi gases and showed that the BCS-Leggett theory can be reproduced as the leading-order large-N theory^49^, but applying the large-N theory to polarized Fermi gases or including the next-order corrections has been a daunting challenge. There have been recent numerical studies using the functional renormalization group (FRG) method to study polarized Fermi gases^50,51^. If the method can be applied to transport phenomena in the future, it may offer more quantitative predictions. Here, we use a t-matrix theory consistent with the BCS-Leggett ground state^41^ and summarize it in the Methods section. While the equations of state are formally the same as Eq. (1), one has to distinguish the order parameter Δ_*sc*_ from the total gap ${\rm{\Delta }}=\sqrt{{{\rm{\Delta }}}_{sc}^{2}+{{\rm{\Delta }}}_{pg}^{2}}$ with the contribution Δ_*pg*_ from the non-condensed pairs. Similar to the mean-field result, the polarized Fermi gas at low temperatures is unstable against phase separation in the BCS and unitary regimes^28,32^.

**Figure 1:**
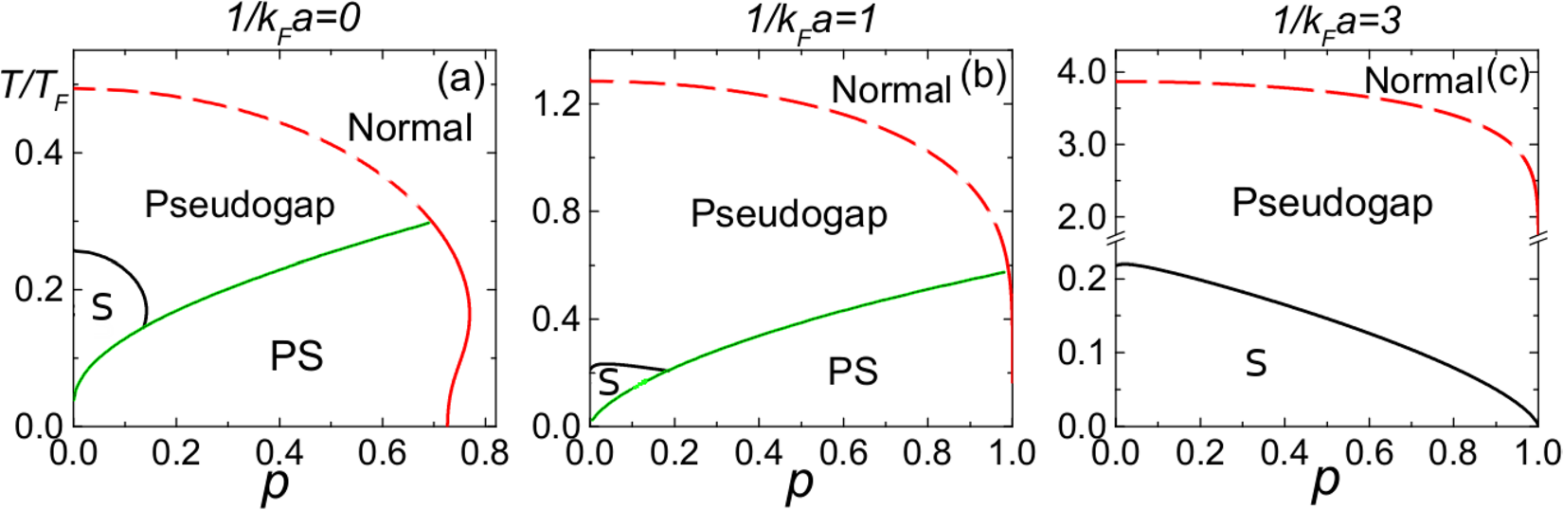
*T*-*p* phase diagrams of population-imbalanced Fermi gases in a box potential (**a**) in the unitary limit (1/*k*_*F*_*a* = 0) and (**b**), (**c**) in the BEC regime (1/*k*_*F*_*a* = 1, 3). ‘S’ indicates the uniform superfluid phase, ‘PS’ corresponds to the phase separation, ‘Pseudogap’ denotes the homogeneous paired normal phase, and ‘Normal’ denotes the unpaired normal phase. There is no phase transition between the pseudogap and unpaired normal phases. Here $n={k}_{F}^{3}\mathrm{/3}{\pi }^{2}$, ${E}_{F}={k}_{B}{T}_{F}={\hslash }^{2}{k}_{F}^{2}\mathrm{/2}m$.

The phase diagrams of polarized Fermi gases have been shown in refs^32,42^. for box potentials and harmonic traps. We summarize again in Fig. 1 the phase diagrams of polarized Fermi gases in a box potential in the unitary ((a) for 1/(*k*_*F*_*a*) = 0) and BEC ((b) for 1/(*k*_*F*_*a*) = 1 and (c) for 1/(*k*_*F*_*a*) = 3) regimes. The polarization is defined by *p* = *δn*/*n*. The homogeneous superfluid or pseudogap phase in the BCS and unitary regimes is unstable at low temperatures against phase separation (PS)^48^. Since we focus on the shear viscosity of homogeneous Fermi gases, we will address the regimes besides the PS regions shown in Fig. 1 and leave the definition and discussion of the viscosity in the PS regime for future work. We caution that Fig. 1, based on a particular t-matrix theory, only captures qualitative features. For unpolarized unitary Fermi gases, the superfluid transition temperature measured in experiment is around 0.16*T*_*F*_^7^ and the t-matrix theory overestimates it. Moreover, the experimental value^52^ of critical polarization where superfluidity no longer survives in the ground state of polarized unitary Fermi gases is about the same value of 0.7 shown in Fig. 1(a). However, the background harmonic trap may have increased the experimental value^48^.

### Shear viscosity and anomalous shear viscosity of polarized Fermi superfluids

The shear viscosity can be obtained from the energy-momentum tensor in linear response theory, and it can be simplified by using the current-current response function^43,53^. We summarize the connection in the Supplemental Information (SI). Explicitly,

2 $$\eta =-{m}^{2}\mathop{\mathrm{lim}}\limits_{\omega \to 0}\,\mathop{\mathrm{lim}}\limits_{q\to 0}\frac{\omega }{{q}^{2}}{\rm{I}}{\rm{m}}{K}_{{\rm{T}}}(\omega ,{\bf{q}}),$$

where the current-current response function and its transverse part *K*_T_ can be obtained from the gauge-invariant CFOP theory summarized in the SI. Previous studies of the shear viscosity of superfluid helium-3 used a kinetic-equation approach, where gauge-invariance has also been carefully addressed^21^. In the unitary and BEC regimes, the shear viscosity of strongly interacting Fermi gases receives contributions from the condensed and noncondensed fermion pairs and fermionic quasiparticles. Thus, ***η*** = ***η***_f_ + ***η***_b_ with the subscripts “f” and “b” representing the condensed-pair (plus fermionic-quasiparticle) and noncondensed-pair contributions. To ensure the consistency between the thermodynamic quantities and response functions, a gauge-invariant vertex satisfying the Ward identity needs to be constructed, as summarized in the SI.

When the attractive interaction becomes stronger, finding an explicit expression of a gauge invariant vertex is difficult since the vertex must be modified in the same way as the self-energy in the Green’s function^54^. After incorporating the relaxation time *τ* of the current-current response function from linear response theory, the fermionic part of the shear viscosity becomes

3 $${\eta }_{{\rm{f}}}=\frac{1}{30{\pi }^{2}{m}^{2}}{\int }_{0}^{\infty }dk{k}^{6}\,(1-\frac{{{\rm{\Delta }}}_{{\rm{pg}}}^{2}}{{E}_{{\bf{k}}}^{2}})\frac{{\xi }_{{\bf{k}}}^{2}}{{E}_{{\bf{k}}}^{2}}[-\frac{\partial f({E}_{{\bf{k}}\uparrow })}{\partial {E}_{{\bf{k}}\uparrow }}-\frac{\partial f({E}_{{\bf{k}}\downarrow })}{\partial {E}_{{\bf{k}}\downarrow }}]\tau \mathrm{.}$$

We emphasize that Eq. (3) also contains contributions from the bosonic excitations (non-condensed pairs) via the terms involving ${{\rm{\Delta }}}_{{\rm{pg}}}^{2}$, which reflect a reduction of the fermionic normal fluid due to strong pairing effect. The bosonic contribution *η*_b_ comes from the noncondensed pairs approximated by a noninteracting Bose gas with renormalized mass and chemical potential in our theory. The pseudogap is approximated by ${{\rm{\Delta }}}_{{\rm{pg}}}^{2}\approx {a}_{0}^{-1}\,{\sum }_{{\bf{q}}}b({{\rm{\Omega }}}_{{\bf{q}}})$, where *b*(*x*) = 1/(exp(*x*/*T*) −1) is the Bose distribution function, and the coefficient ${a}_{0}^{-1}$ and dispersion Ω_***q***_ are given in the Methods. Hence,

4 $${\eta }_{b}=-\frac{1}{30{\pi }^{2}{M}^{\ast 2}}{\int }_{0}^{\infty }dk{k}^{6}\frac{\partial b({{\rm{\Omega }}}_{{\bf{k}}})}{\partial {{\rm{\Omega }}}_{{\bf{k}}}}\tau \mathrm{.}$$

The derivations of *η*_*f*_ and *η*_*b*_ can be found in the Supplemental Information. When the polarization vanishes, the expressions reproduce the results of ref.^43^.

As pointed out in ref.^43^, for the BCS superfluid and its generalizations, there is an additional contribution to the shear viscosity coming from the transverse momentum transfer via the Cooper pairs. This is called the anomalous shear viscosity *χ*. While *η* can be found from the energy-momentum stress tensor, *χ* arises from the anomalous stress tensor $$\overleftrightarrow{{\rm{\Pi }}}({\bf{x}})=\frac{1}{m}(\nabla {\psi }_{\downarrow }({\bf{x}})\nabla {\psi }_{\uparrow }({\bf{x}})+\nabla {\psi }_{\uparrow }^{\dagger }({\bf{x}})\nabla {\psi }_{\downarrow }^{\dagger }({\bf{x}}))$$. The anomalous shear viscosity is then obtained from the $$\overleftrightarrow{{\rm{\Pi }}}$$ − $$\overleftrightarrow{{\rm{\Pi }}}$$ response function via $$\chi \equiv -{\mathrm{lim}}_{\omega \to 0}{\mathrm{lim}}_{q\to 0}\frac{1}{\omega }{\rm{Im}}[{Q}^{xyxy}(\omega ,{\bf{q}})]$$. Here $$\overleftrightarrow{Q}(\bar{\tau }-\bar{\tau }\text{'},{\bf{q}})=$$$$-i\theta (\bar{\tau }-\bar{\tau }\text{'})\langle [\overleftrightarrow{{\rm{\Pi }}}(\bar{\tau },{\bf{q}}),\overleftrightarrow{{\rm{\Pi }}}(\bar{\tau }\text{'},-{\bf{q}})]\rangle $$ with $$\bar{\tau }$$ being the imaginary time and *θ* (*x*) the Heaviside step function. The experimentally measured viscosity may be considered as the sum of *η* and *χ*^43^.

There exists a relation for unpolarized unitary Fermi superfluids connecting some thermodynamic quantities and transport coefficients^43^. At the mean-field level when the polarization has been included in the equations of motion and linear response theory, we found the following relation

5 $$\eta +\chi =(P-\frac{2}{5}\mu {n}_{s})\tau \mathrm{.}$$

Here *η* is obtained from the CFOP theory and its expression is similar to *η*_f_, except the total gap Δ plays the role of the order parameter Δ_*sc*_, *χ* is the anomalous shear viscosity representing the momentum transfer via the Cooper pairs, *P* is the pressure, *n*_*s*_ is the superfluid density, and *τ* is the relaxation time. A derivation of the exact relation and the expressions of the quantities involved are given in the Supplemental Information. The relation also shows the consistency between the equations of state and linear response theory implemented here, and it naturally reproduces the relation of unpolarized unitary Fermi gases^43^. We also note that while the relation *η* = *n*_*n*_*D*^44^ is an Einstein relation between a transport coefficient from a response function and a susceptibility from a response function. The relation (5) does not have such a structure.

In the presence of pairing fluctuations, the exact relation corresponding to Eq. (5) has not been fully resolved and an approximate relation was proposed instead^43^. Here we follow a similar idea and construct an approximate relation. A natural generalization is to include the contribution from the noncondensed bosons. However, the anomalous shear viscosity mainly measures the momentum transfer through the Cooper pairs and we do not include the pairing fluctuations there. Thus,

6 $$\chi \approx -\frac{1}{15}\sum _{{\bf{k}}}\frac{{k}^{4}}{{m}^{2}}\frac{{{\rm{\Delta }}}_{{\rm{sc}}}^{2}}{{E}_{{\bf{k}}}^{2}}(\frac{\partial f({E}_{{\bf{k}}\uparrow })}{\partial {E}_{{\bf{k}}\uparrow }}+\frac{\partial f({E}_{{\bf{k}}\downarrow })}{\partial {E}_{{\bf{k}}\downarrow }})\tau \mathrm{.}$$

An approximate relation for unitary Fermi superfluids with population imbalance beyond the mean-field theory is proposed here:

7 $$\eta +\chi \approx (P-\frac{2}{5}\mu n)\tau \mathrm{.}$$

Here *η* = *η*_f_ + *η*_b_, *P* = *P*_f_ + *P*_b_. The expressions of the involved quantities can be found in the Supplemental Information.

Here we have assumed the relaxation time of the composite bosons is the same as that of the fermions since they are in local equilibrium. We caution that the interactions between the fermions may be different from those between the pairs^55^, and a full treatment may introduce corrections to our approximation. For BCS superfluids, the relaxation time *τ* may be obtained by the Boltzmann equation approach of the Bogoliubov quasi-particles^21^, and a similar approach has been applied to unpolarized unitary Fermi gases at high temperatures^56–58^. Another approach is to use the approximate relation *τ* ≈ −2ImΣ(*K*)^3^, where Σ is the self-energy of fermionic quasi-particles. Experimental measurements of *τ* of ultracold atoms usually rely on hydrodynamic relations^6,20^ and are further complicated by the inhomogeneity from the harmonic trap.

**Figure 2:**
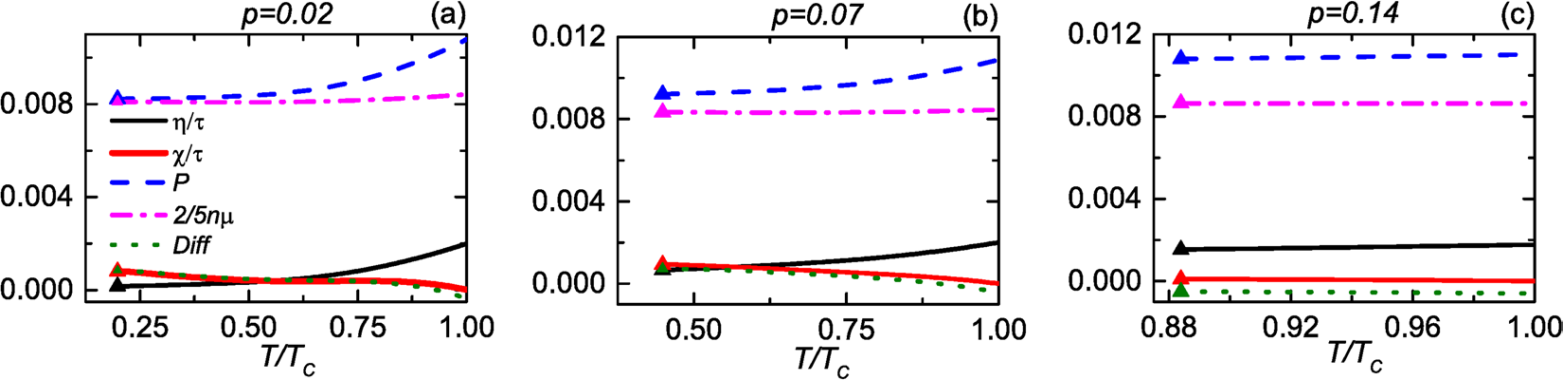
*η*/*τ* (black line), *χ*/*τ* (red line), pressure *P* (blue dashed line), $\frac{2}{5}n\mu$ (pink dot-dash line) and ${\rm{Diff}}\equiv (\eta +\chi )/\tau +\frac{2}{5}n\mu -P$ (green dotted line) of a unitary Fermi gas as a function of temperature (below *T*_*c*_) for *p* = 0.02 (**a**), 0.07 (**b**) and 0.14 (**c**). The unit of *η*/*τ* and the other quantities is ${E}_{F}{k}_{F}^{3}$. The triangles label where phase separation occurs.

We use numerical calculations to check the validity of our approximation and present a comparison in Fig. 2, where we show *η*/*τ*, *χ*/*τ*, *P*, and $\frac{2}{5}n\mu$ as a function of *T*/*T*_*c*_ for *p* = 0.02, 0.07, and 0.14. The corresponding *T*_*c*_/*T*_*F*_ values are 0.262, 0.252, and 0.198, and we caution that it is known the *t*-matrix overestimates *T*_*c*_^37^. The deviation from the identity (7), called ${\rm{Diff}}\equiv (\eta +\chi )/\tau +\frac{2}{5}n\mu -P$, is also shown in Fig. 2. The largest relative error, defined by |Diff/*P*|, is 7.4% for *p* = 0.02, 7.8% for *p* = 0.07, and 5.5% for *p* = 0.14, respectively. Hence, the approximate relation works reasonably.

### Temperature and interaction effects on the viscoisty of polarized Fermi superfluids

A first-principle evaluation of *τ* below *T*_*c*_ for polarized unitary Fermi superfluids remains a challenge mainly because the conventional kinetic-equation method does not apply to such a complex situation. On the other hand, the ratio *η*/*τ* already contains information from thermodynamics via the equations of motion and from transport via the linear response. Moreover, *η*/*τ* and *χ*/*τ* have the dimension of energy density *E*/*V* (which is the same as the dimension of pressure *P*), and we have shown there is a relation connecting them to certain thermodynamic quantities and transport coefficients. Finally, *η*/*τ* and *χ*/*τ* can be directly obtained from linear response theory. Therefore, here we focus on the behavior of (*η* + *χ*)/*τ*.

**Figure 3:**
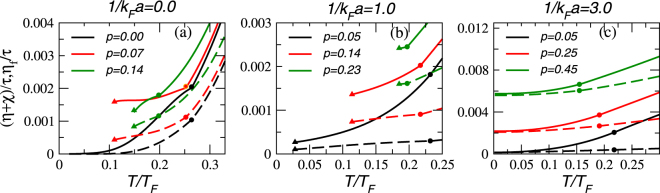
The ratio of the total shear viscosity *η* + *χ* and relaxation time *τ* (in units of ${E}_{F}{k}_{F}^{3}$) as a function of temperature at unitarity (**a**) and the BEC side (**b**) for different *p*. The triangles denote where the homogeneous phase becomes unstable and phase separation occurs. The circle denotes where *T*_*c*_ is. The solid lines correspond to *η*/*τ*, and the dashed lines correspond to *η*_*f*_/*τ*.

In Fig. 3, (*η* + *χ*)/*τ* and are plotted as a function of temperature for polarized Fermi gases in the unitary and BEC regimes with selected values of the polarization *p*. In the unitary limit shown in panel (a), the polarization is restricted to $0\le p\lesssim 0.14$, where superfluidity exists (see Fig. 1(a)). Both (*η* + *χ*)/*τ* and *η*_f_/*τ* increase with temperature since the number of condensed pairs decreases due to the proliferation of thermal excitations. We found that the non-condensed pair contribution *η*_b_/*τ* also increases with temperature since the number of non-condensed pairs, which are treated as a normal bosonic gas, increases with *T* below the pairing onset temperature *T* ^*^. In the BEC regime as illustrated in panel (b), the polarization is restricted to $p\lesssim 0.21$, where superfluidity survives at low temperatures. Only when $\mathrm{1/}{k}_{F}a\gtrsim 2.3$ in the deep BEC regime, the system becomes fully stable against phase separation. Panel (c) shows the case of 1/*k*_*F*_*a* = 3.0 and the uniform superfluid phase is stable at low temperatures. Although the basic trend in (c) is similar to panel (b), *η*_b_/*τ* contributes more significantly as temperature increases. This is because the noncondensed pairs in the deep BEC regime behave like thermal bosons, whose fraction increases with temperature.

**Figure 4:**
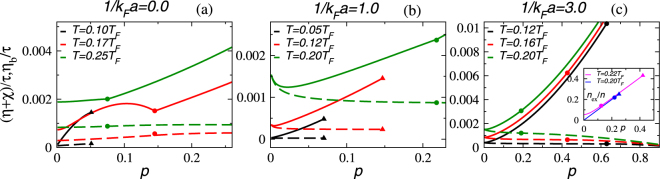
The ratio of the total shear viscosity *η* + *χ* and relaxation time *τ* (in units of ${E}_{F}{k}_{F}^{3}$) as a function of *p* in the unitary limit (**a**), BEC side (**b**) and deep BEC side (**c**) at different temperatures. The convention is the same as Fig. 3 except the dashed lines denote *η*_*b*_/*τ*. The triangles denote where the homogeneous phase becomes unstable and phase separation occurs. The circle denotes where *T*_*c*_ is. The inset of panel (c) shows the number fraction of gapless excitations, *n*_*ex*_/*n*, as a function of *p*. The longer (shorter) line indicates the situation with *T* = 0.22*T*_*F*_ and 1/(*k*_*F*_*a*) = 0 at unitarity (with *T* = 0.20*T*_*F*_ and 1/(*k*_*F*_*a*) = 1 in the BEC regime).

To better understand the dependence of the shear viscosity on the polarization, we show in Fig. 4 (*η* + *χ*)/*τ* and *η*_b_/*τ* as a function of *p* at selected temperatures in the unitary and BEC regimes. The increase of (*η* + *χ*)/*τ* with *p* is because a population-imbalanced Fermi superfluid is a homogeneous mixture of the condensed pairs and excess majority fermions. The excess fermions cause gapless excitations acting like a normal fluid, which in turn leads to the finite shear viscosity. (This will be elaborated below). Therefore, as the polarization increases, the relative population of condensed pairs decreases, and the shear viscosity increases.

The competitions among the excess fermions, condensed pairs, fermion quasiparticles, and noncondensed pairs may lead to non-monotonic behavior of (*η* + *χ*)/*τ* in several instances. The contribution of *χ*/*τ* at unitarity and intermediate temperatures can show a dome-shape dependence on *p*. This is because increasing *p* increases the number of excess fermions (and increases *η*_*f*_) but decreases the number of Cooper pairs (and decreases *χ*). When the two effects are comparable, there is a local maximum as *p* increases. For the unitary case at higher temperatures, the decrease in *χ* is relatively minor when compared to the increase in *η*_*f*_, so the overall viscosity behaves monotonically.

On the other hand, the noncondensed pair contribution *η*_*b*_/*τ* at unitarity and low temperatures shows a relatively upward trend as *p* increases but saturates at higher temperatures. This trend is opposite to that in the BEC regimes, shown in panels (b) and (c) because the pairing gap at unitarity is smaller compared to the gap in the deep BEC regime. Therefore, the condensed and noncondensed pairs are more sensitive to temperature and polarization at unitarity. In the deep BEC regime, the effective mass of pairs, M^*^, approaches 2*m* because the fermions are tightly bound. However, we found M^*^ increases with *p* at unitarity instead. In Eq. (4), M^*^ appears both in the denominator and the bosonic dispersion Ω_**q**_. The combined effects cause the upward trend of *η*_b_/*τ* as *p* increases at low temperatures in the unitary limit. In the BEC regime, M^*^ no longer increases with *p*, so *η*_b_/*τ* decreases with *p* due to a decreasing fraction of the paired fermions.

Figure 4(b) shows the results in the shallow BEC regime with 1/*k*_*F*_*a* = 1.0. Here, the noncondensed pairs contribute significantly to the shear viscosity at low *p*. Indeed, $(\eta +\chi )\simeq {\eta }_{b}$ at low *p* because the condensed pairs form a superfluid and do not contribute to the shear viscosity and *χ*/*τ* is small, therefore the contribution is mostly from the noncondensed pairs behaving like a normal fluid. At higher temperatures, interestingly, *η*/*τ* is not monotonic as *p* increases and a minimum emerges. This is because the number of fermion pairs, including both the condensed and noncondensed pairs, decreases as *p* or *T* increases as more excess fermions or fermionic quasiparticles are present. Hence, *η*_b_/*τ* starts to decrease with *p* at higher temperatures. Meanwhile, the fraction of excess majority fermions increases with *p*, and they also increase the shear viscosity. The excess fermions do not participate in pairing and they occupy certain regions in momentum space^48^. In the shallow BEC regime illustrated in Fig. 4(b), a competition between a suppression of the non-condensed pairs and an increase of the excess-fermions causing gapless excitations as *p* increases leads to a minimum in (*η* + *χ*)/*τ* at intermediate polarization and temperature. It may be challenging to determine the maximum or minimum of (*η* + *χ*)/*τ* experimentally because the relaxation time *τ* is not easily measurable, but observing the non-monotonic behavior could demonstrate the competitions among different mechanisms of the viscosity of polarized Fermi gases.

Figure 4(c) shows the result in the deep BEC regime. Since the strongly attractive interactions allow the superfluid and pseudogap phases to be highly polarized and accommodate the excess fermions, the bosonic contribution *η*_*b*_ becomes less dominant as *p* increases. Moreover, thermal excitations are also less prominent because both the noncondensed pairs and excess fermions have smooth thermal distributions. The shear viscosity of polarized Fermi gases comes mainly from the gapless excitations caused by the excess fermions. This can be understood by the energy dispersion ${E}_{{\bf{k}}\uparrow ,\downarrow }={E}_{{\bf{k}}}\,\mp \,h$ of the fermionic excitations. When *n*_↑_ > *n*_↓_, *h* > 0 and *E*_**k**_ < 0 if *k* ∈ [*k*_1_, *k*_2_] and *μ*^2^ + Δ^2^ ≥ *h*^2^ or if *k* ∈ [0, *k*_2_] and *μ*^2^ + Δ^2^ < *h*^2^, where ${k}_{\mathrm{1,}2}=\sqrt{2m\mu \mp 2m\sqrt{{h}^{2}-{{\rm{\Delta }}}^{2}}}$. In both cases, the excitations are gapless because of the excess fermions. The contribution from the gapless excitations can be estimated by *n*_*ex*_ = ∑_**k**__,__*σ*_*f*(*E*_**k**_, _*σ*_), which only takes significant values if the dispersion is gapless and the number of fermionic excitations is large. In both unitary and BEC regimes, *n*_ex_ increases with *p* as shown in the inset of Fig. 4(c). The fermionic excitations behave like a normal fluid and dominate the contribution to the shear viscosity at higher *p*. Thus, in the deep BEC regime (*η* + *χ*)/*τ* is monotonic as *p* increases.

## Discussion

The shear viscosity of homogeneous, population-imbalanced atomic Fermi gases in the unitary limit and BEC regimes has been analyzed, although phase separation at low temperatures in the BCS and unitary regimes hinders a full description. The contributions from noncondensed pairs are included by a pairing-fluctuation theory applicable to polarized Fermi gases in the BCS-BEC crossover. In general, the ratio between the total shear viscosity and relaxation time increases with temperature and polarization, but competitions between the condensed and noncondensed pairs, fermionic quasiparticles, and excess fermions may lead to local maximum or minimum of (*η* + *χ*)/*τ* in the unitary limit or shallow BEC regimes as *p* increases. This is our first message of the work.

The relation for polarized unitary Fermi superfluids connects certain thermodynamic quantities and transport coefficients. It also helps determine the relaxation time and constrain the physical quantities. Moreover, the anomalous shear viscosity from the Cooper pairs should be included. This is our second message. The relation is exact at the mean-field level, but the presence of pairing fluctuations only allows for a proposed approximation. The full expression of the relation awaits future investigations. Our results offer qualitative pictures of the shear viscosity of polarized Fermi gases in the unitary and strongly attractive regimes. Recent progress on measuring thermodynamic quantities in homogeneous unpolarized^59^ and polarized^60^ Fermi gases may eventually verify the relation and check the value of *τ*.

## Methods

### Beyond mean-field theory of polarized Fermi gases

As the attractive coupling *g* increases, preformed pairs not contributing to the superfluid start to form at finite temperatures. We consider the more realistic situation which includes the pairing fluctuation effects^5,37,38,40,41^. Here, we follow a particular scheme^32,37^ consistent with the BCS-Leggett ground state in the unpolarized limit. The full Green’s function is *G*_*σ*_(*K*) = [*G*_0*σ*_(*K*) − Σ_*σ*_(*K*)]^−1^. Here *G*_0*σ*_(*K*) = (*iω*_*n*_ − *ξ*_***k****σ*_)^−1^ is the bare Green’s function with *ξ*_**k***σ*_ = *ε*_**k***σ*_ − *μ*_*σ*_ and the fermion self-energy is ${{\rm{\Sigma }}}_{\sigma }(K)={\sum }_{Q}t(Q){G}_{0\bar{\sigma }}(Q-K)$ with $\bar{\sigma }$ being the opposite of *σ*. To construct the *t*-matrix, we consider a spin-symmetrized pair susceptibility, or one rung of the ladder diagrams, consisting of one bare and one full Green’s functions: $X(Q)=\frac{1}{2}{\sum }_{K}[{G}_{0\uparrow }(Q-K){G}_{\downarrow }(K)+{G}_{0\downarrow }(Q-K){G}_{\uparrow }(K)]$. The *t*-matrix *t*(*Q*) is separated into the condensed (sc, *Q* = 0) and noncondensed (pg, *Q* ≠ 0) pair contributions: *t*(*Q*) ≈ *t*_sc_ + *t*_pg_ with ${t}_{{\rm{s}}{\rm{c}}}(Q)=-({\Delta }_{{\rm{s}}{\rm{c}}}^{2}/T)\delta (Q)$ and *t*_*pg*_(*Q*) = [*g*^−1^ + *X*(*Q*)]^−1^. Then, the total gap has the form Δ^2^(*T*) = Δ^2^_sc_(*T*) + Δ^2^_pg_(*T*). Here Δ_*sc*_ is the order parameter and Δ_pg_ is the pseudogap which is approximated by ${{\rm{\Delta }}}_{{\rm{pg}}}^{2}\approx -{\sum }_{Q\ne 0}{t}_{{\rm{pg}}}(Q)$. The pairing onset temperature T^*^ indicates where the total gap Δ vanishes, while the superfluid transition temperature *T*_*c*_ indicates where the order parameter Δ_sc_ vanishes. The equations of state can be derived from the Green’s functions by *n* = ∑_*K*,*σ*_*G*_*σ*_(*K*), *δn* = ∑_*K*_(*G*_↑_ (*K*) − *G*_↓_ (*K*)) and $\frac{1}{g}=\frac{1}{2}{\sum }_{K,\sigma }{G}_{\sigma }(K){G}_{0\bar{\sigma }}(-K)$. For our numerical calculations, the *t*-matrix is approximated by^61^ ${t}_{{\rm{pg}}}({\rm{\Omega }},{\bf{q}})\approx \frac{1}{{a}_{0}(\omega -{{\rm{\Omega }}}_{{\bf{q}}})}$. Here ${a}_{0}=\frac{{\rm{\partial }}\chi (Q)}{{\rm{\partial }}{\rm{\Omega }}}{|}_{Q=0}$ and Ω_**q**_ = (*q*^2^)/(2M^*^) − *μ*_pair_ with ${M}^{\ast }=12\frac{{{\rm{\partial }}}^{2}\chi (Q)}{{\rm{\partial }}{q}^{2}}{|}_{Q=0}$ being the effective pair mass and *μ*_*pair*_ ≤ 0 the pair chemical potential.

To locate where phase separation emerges at low temperatures, we adopt a simplified approach: the unpaired normal phase has a fraction *x* of the total particle density while the paired phase has a fraction 1 − *x*, and the two phases are separated by an interface with positive energy. Since the system is in equilibrium, *T*, *μ*_*σ*_, and *P* should be continuous across the interface^42,62^. The boundary between a stable polarized superfluid phase or pseudogap phase and the phase separation is given by the condition *x* = 0. In the deep BEC regime, the pairing gap is large and the polarized superfluid phase is robust, therefore no phase separation occurs at low temperatures.

### Data availability

All data generated or analysed during this study are included in this published article (and its Supplementary Information files).

## Electronic supplementary material

Supplementary Information
