# Supplementary material for: Shear Viscosity of Uniform Fermi Gases with Population Imbalance
Source: Sci Rep. 2018 Mar 5;8:3981. doi: 10.1038/s41598-018-22273-1 (PMC5838242; doi:10.1038/s41598-018-22273-1)
Supplement: Supplementary file 1 — Supplementary Information [file 41598_2018_22273_MOESM1_ESM.pdf]

Supplmental Information: Shear Viscosity of Uniform Fermi Gases with Population Imbalance

Weimin Cai[1], Yan He[2], Hao Guo[1], Chih-Chun Chien[3]

[1] *Department of Physics, Southeast University, Nanjing 211189, China*

[2] *College of Physical Science and Technology, Sichuan University, Chengdu, Sichuan 610064, China and*

[3] *School of Natural Sciences, University of California, Merced, CA 95343, USA**

I. SHEAR VISCOSITY FROM GAUGE-INVARIANT LINEAR RESPONSE THEORY

The shear viscosity can be obtained from the energy-momentum stress tensor response function [1]:

$$\eta = -\lim_{\omega\to 0}\lim_{q\to 0}\frac{1}{\omega}\mathrm{Im}Q^{xyxy}_{\mathrm{TT}}(\omega,\mathbf{q}), \tag{1}$$

where $\overleftrightarrow{Q}_{\mathrm{TT}}$ is the stress tensor-stress tensor response function

$$\overleftrightarrow{Q}_{\mathrm{TT}}(\tau-\tau',\mathbf{q}) = -i\theta(\tau-\tau')\langle\overleftrightarrow{T}(\tau,\mathbf{q}),\overleftrightarrow{T}(\tau',-\mathbf{q})\rangle. \tag{2}$$

Here T^{ij} is the spatial part of the energy-momentum stress tensor satisfying the momentum flux conservation law

$$\frac{\partial T^{0j}}{\partial t} + \frac{\partial T^{ij}}{\partial x^i} = 0, \tag{3}$$

where $T^{0i} = mJ^i$ and J^i is the particle current. Hence we can apply this identity to replace the stress tensor-stress tensor response function by the current-current response function K^{ij} discussed below (see Ref. [2] for details). Explicitly,

$$\eta = -m^2\lim_{\omega\to 0}\lim_{q\to 0}\frac{\omega^2}{q^2}\mathrm{Im}K_{\mathrm{T}}(\omega,\mathbf{q}). \tag{4}$$

In the following we show how to derive K^{ij} and its transverse part K_{T}.

For population-imbalanced Fermi gases, the Hamiltonian respects a global U(1) symmetry $\psi_\sigma \to e^{-i\alpha}\psi_\sigma$, where ψ_σ is the fermionic field for each species. The current-current response function is evaluated from a gauge invariant linear response theory [3], which can be obtained by "gauging" the U(1) symmetry. To implement it, the symmetry becomes a local symmetry and we introduce an effective gauge field to maintain the symmetry. The gauge field, which can be thought of as an effective electromagnetic (EM) field $A^\mu = (\phi,\mathbf{A})$, interacts with the fermionic field by coupling with the Noether current of the U(1) symmetry given by $J^\mu = (n,\mathbf{J})$. Here

$$\mathbf{J}(\mathbf{x}) = -\frac{1}{2mi}\sum_\sigma\Big[\psi^\dagger_\sigma(\mathbf{x})\big(\nabla\psi_\sigma(\mathbf{x})\big) - \big(\nabla\psi^\dagger_\sigma(\mathbf{x})\big)\psi_\sigma(\mathbf{x})\Big]\frac{1}{m}\mathbf{A}(\mathbf{x})\sum_\sigma\psi^\dagger_\sigma(\mathbf{x})\psi_\sigma(\mathbf{x}),\ n(\mathbf{x}) = \sum_\sigma\psi^\dagger_\sigma(\mathbf{x})\psi_\sigma(\mathbf{x}). \tag{5}$$

The conserved current is perturbed by the effective external EM field as $\delta J^\mu(Q) = K^{\mu\nu}A_\nu(Q)$, where δJ^μ is the perturbed mass current, and

$$K^{\mu\nu}(Q) = \frac{n}{m}h^{\mu\nu} + \sum_{K\sigma}\Gamma^\mu_\sigma(K+Q,K)G_\sigma(K+Q)\gamma^\nu_\sigma(K,K+Q)G_\sigma(K) \tag{6}$$

is the EM response function. Here $\gamma^\mu_\sigma(K+Q,K) = S_\sigma(1,\frac{\mathbf{p}+\frac{\mathbf{q}}{2}}{m})$ and $S_{\uparrow,\downarrow} = \pm 1$ is the bare EM interaction vertex, $\Gamma^\mu_\sigma(K+Q,K)$ is the full EM interaction vertex and $h^{\mu\nu} = -\eta^{\mu\nu}(1-\eta^{\nu 0})$ with $\eta^{\mu\nu} = \mathrm{diag}(1,-1,-1,-1)$ being the metric tensor. The transverse current-current response function is defined by $K_{\mathrm{T}} = (\sum_{i=x}^{z}K^{ii}_{\mathrm{JJ}} - K_{\mathrm{L}})/2$ with the longitudinal part given by $K_{\mathrm{L}} = \hat{\mathbf{q}}\cdot\overleftrightarrow{K}_{\mathrm{JJ}}\cdot\hat{\mathbf{q}}$. The frequency is obtained by a complex continuation of the bosonic Matsubara frequency $i\Omega_l \to \omega + i0^+$, so Q becomes $(\omega,\mathbf{q})$ and $\hat{\mathbf{q}} = \mathbf{q}/|\mathbf{q}|$.

---

* guohao.ph@seu.edu.cn; cchien5@ucmerced.edu

In a gauge invariant theory, the vertex must satisfy the Ward identity [4–6]

$$q_\mu \Gamma^\mu_\sigma(K+Q,K) = G^{-1}_\sigma(K+Q) - G^{-1}_\sigma(K). \tag{7}$$

It will guarantee that the perturbed current is also conserved: $q_\mu \delta J^\mu(Q) = 0$. The gauge invariant EM vertex and the response function $\overleftrightarrow{K}$ for unpolarized Fermi gases within the BCS mean field formalism can be found in Ref. [3].

The current-current response functions correspond to the spatial part of Eq. (6) and can be decomposed in to the form

$$\overleftrightarrow{K} = \overleftrightarrow{\mathcal{P}} + \frac{\overleftrightarrow{n}}{m} + \overleftrightarrow{C}, \tag{8}$$

where $\overleftrightarrow{n} = n\overleftrightarrow{1}$ with $\overleftrightarrow{1}$ being the unit tensor has no imaginary part and gives no contribution to the shear viscosity, and $\overleftrightarrow{C}$ comes from the contributions of collective modes and does not contribute to the shear viscosity [7]. Only the paramagnetic response function $\overleftrightarrow{\mathcal{P}}$ is relevant and its expression is given in Sec. II.

Within the pairing fluctuation formalism consistent with the Leggett-BCS theory [7, 9], a gauge invariant EM vertex respecting the Ward identity has the form

$$\begin{aligned}\Gamma^\mu_\sigma(K+Q,K) &= \gamma^\mu_\sigma(K+Q,K) + \Gamma^\mu_{\text{Coll},\sigma}(K+Q,K)\\ &+ \Gamma^\mu_{\text{MT,sc},\sigma}(K+Q,K) + \Gamma^\mu_{\text{MT,pg},\sigma}(K+Q,K) + \Gamma^\mu_{\text{AL},1,\sigma}(K+Q,K) + \Gamma^\mu_{\text{AL},2,\sigma}(K+Q,K).\end{aligned} \tag{9}$$

The second term $\Gamma^\mu_{\text{Coll},\sigma}(K+Q,K)$ in the expression stands for the contributions from the collective modes due to the spontaneous breaking of the U(1) symmetry in the superfluid phase. However, this term is irrelevant when we derive the shear viscosity [6], so we skip its full expression. The third and fourth terms come from the Maki-Thompson (MT) diagrams associated with the condensed and non-condensed pairs, respectively, and the fifth and sixth terms are two Aslamazov-Larkin (AL) diagrams introduced in a way satisfying the Ward identity. The expressions of those diagrams can be found in Sec. III. By using the identity (27), the paramagnetic response function is given by Eq. (28). It can be proven that this formalism satisfies the sum rule [1, 10]

$$\lim_{\mathbf{q}\to 0}\int_{\infty}^{\infty}\Big(-\frac{\text{Im}K_{\text{T}}(\omega,\mathbf{q})}{\omega}\Big) = \frac{n_{\text{n}}(T)}{m}. \tag{10}$$

Here $n_{\text{n}}(T) = n - n_s(T)$ is the normal-fluid density. All the expressions apply to homogeneous population-imbalanced Fermi gases when the corresponding thermodynamic quantities are used.

II. DETAILS OF MEAN-FIELD THEORY

By defining $E^\pm_{\mathbf{k}} = E_{\mathbf{k}\pm\frac{\mathbf{q}}{2}}$, $E^\pm_{\mathbf{k}\downarrow,\uparrow} = E^\pm_{\mathbf{k}} \mp h$ and letting $K^{\mu\nu} = Q^{\mu\nu} + \frac{n}{m}h^{\mu\nu}$, the paramagnetic current-current response function can be derived from Eq. (8). The expression is

$$\begin{aligned}\overleftrightarrow{\mathcal{P}}^{ij}(\omega,\mathbf{q}) = \sum_{\mathbf{k}}\frac{\mathbf{k}^i\mathbf{k}^j}{2m^2}\Big\{&\Big(1-\frac{\xi^+_{\mathbf{k}}\xi^-_{\mathbf{k}}+\Delta^2}{E^+_{\mathbf{k}}E^-_{\mathbf{k}}}\Big)\Big(\frac{1-f(E^+_{\mathbf{k}\uparrow})-f(E^-_{\mathbf{k}\downarrow})}{\omega-E^+_{\mathbf{k}\uparrow}-E^-_{\mathbf{k}\downarrow}} - \frac{1-f(E^+_{\mathbf{k}\downarrow})-f(E^-_{\mathbf{k}\uparrow})}{\omega+E^+_{\mathbf{k}\downarrow}+E^-_{\mathbf{k}\uparrow}}\Big)\\ &-\Big(1+\frac{\xi^+_{\mathbf{k}}\xi^-_{\mathbf{k}}+\Delta^2}{E^+_{\mathbf{k}}E^-_{\mathbf{k}}}\Big)\Big(\frac{f(E^+_{\mathbf{k}\uparrow})-f(E^-_{\mathbf{k}\uparrow})}{\omega-E^+_{\mathbf{k}\uparrow}+E^-_{\mathbf{k}\uparrow}} - \frac{f(E^+_{\mathbf{k}\downarrow})-f(E^-_{\mathbf{k}\downarrow})}{\omega+E^+_{\mathbf{k}\downarrow}-E^-_{\mathbf{k}\downarrow}}\Big)\Big\}.\end{aligned} \tag{11}$$

The expression of the shear viscosity, Eq. (13), can be found by using Eq. (4). We follow the formalism of Ref. [7] and incorporate the relaxation time τ from the current-current response function in linear response theory [8] by regularizing the δ-function in the expression of the response function with a Lorentzian function

$$\delta(x) = \lim_{\Gamma\to 0}\frac{1}{\pi}\frac{\Gamma}{x^2+\Gamma^2}. \tag{12}$$

After some algebra, the shear viscosity becomes

$$\eta = \frac{1}{30\pi^2 m^2}\int_0^\infty dk k^6\frac{\xi^2_{\mathbf{k}}}{E^2_{\mathbf{k}}}\Big[-\frac{\partial f(E_{\mathbf{k}\uparrow})}{\partial E_{\mathbf{k}\uparrow}} - \frac{\partial f(E_{\mathbf{k}\downarrow})}{\partial E_{\mathbf{k}\downarrow}}\Big]\tau, \tag{13}$$

where $\tau = \frac{1}{\Gamma}$ is the relaxation time.

Next, we derive an expression of the anomalous shear viscosity following Ref. [7]. Using the anomalous stress tensor shown in the main text, the $\overleftrightarrow{\Pi}-\overleftrightarrow{\Pi}$ response function, $\overleftrightarrow{Q}$, of Fermi superfluids with population imbalance can be constructed. After applying the Fourier transform and using Wick's theorem, we get

$$\begin{aligned}\overleftrightarrow{Q}(i\Omega_l,\mathbf{q}) &= T\sum_{i\omega_n}\sum_{\mathbf{k}}\mathrm{Tr}\big(\overleftrightarrow{\gamma}(K,K+Q)\hat{G}(K+Q)\overleftrightarrow{\gamma}(K+Q,K)\hat{G}(K)\big)\\ &= T\sum_{i\omega_n}\sum_{\mathbf{k}}\frac{\mathbf{k}(\mathbf{k}+\mathbf{q})(\mathbf{k}+\mathbf{q})\mathbf{k}}{m^4}\big(2F_{\uparrow\downarrow}(K+Q)F_{\uparrow\downarrow}(K)-G_{\downarrow}(-K-Q)G_{\uparrow}(K)-G_{\uparrow}(K+Q)G_{\downarrow}(-K)\big).\end{aligned} \quad (14)$$

Here the interaction vertex has a dyadic form in the Nambu space, $\overleftrightarrow{\gamma}(K,K+Q)=\frac{\mathbf{k}(\mathbf{k}+\mathbf{q})}{m^2}\sigma_1$ with σ_1 being the first Pauli matrix, $\hat{G}(K)=\begin{pmatrix} G_{\uparrow}(K) & F_{\uparrow\downarrow}(K)\\ F_{\downarrow\uparrow}(-K) & -G_{\downarrow}(-K)\end{pmatrix}$ is the Green's function in the Nambu space [3], and

$$F_{\sigma\bar{\sigma}}(K)=-\frac{\Delta}{(i\omega_n-E_{\mathbf{k}\sigma})(i\omega_n+E_{\mathbf{k}\bar{\sigma}})} \quad (15)$$

is the anomalous Green's function. It has the property $F_{\uparrow\downarrow}(-K)=F_{\downarrow\uparrow}(K)$. After plugging in the expressions of Green's functions and following a complex continuation, we get

$$\begin{aligned}\overleftrightarrow{Q}(\omega,\mathbf{q}) = \sum_{\mathbf{k}}\frac{\mathbf{k}^-\mathbf{k}^+\mathbf{k}^+\mathbf{k}^-}{2m^4}\Big\{&\big(1+\frac{\xi^+_{\mathbf{k}}\xi^-_{\mathbf{k}}-\Delta^2}{E^+_{\mathbf{k}}E^-_{\mathbf{k}}}\big)\Big(\frac{1-f(E^+_{\mathbf{k}\uparrow})-f(E^-_{\mathbf{k}\downarrow})}{\omega-E^+_{\mathbf{k}\uparrow}-E^-_{\mathbf{k}\downarrow}}-\frac{1-f(E^+_{\mathbf{k}\downarrow})-f(E^-_{\mathbf{k}\uparrow})}{\omega+E^+_{\mathbf{k}\downarrow}+E^-_{\mathbf{k}\uparrow}}\Big)\\ &-\big(1-\frac{\xi^+_{\mathbf{k}}\xi^-_{\mathbf{k}}-\Delta^2}{E^+_{\mathbf{k}}E^-_{\mathbf{k}}}\big)\Big(\frac{f(E^+_{\mathbf{k}\uparrow})-f(E^-_{\mathbf{k}\uparrow})}{\omega-E^+_{\mathbf{k}\uparrow}+E^-_{\mathbf{k}\uparrow}}-\frac{f(E^+_{\mathbf{k}\downarrow})-f(E^-_{\mathbf{k}\downarrow})}{\omega+E^+_{\mathbf{k}\downarrow}-E^-_{\mathbf{k}\downarrow}}\Big)\Big\},\end{aligned} \quad (16)$$

where $\mathbf{k}^{\pm}=\mathbf{k}\pm\frac{\mathbf{q}}{2}$. By following the same step of Eq. (31) to incorporate the relaxation time, the anomalous shear viscosity is

$$\chi=-\frac{1}{15}\sum_{\mathbf{k}}\frac{k^4}{m^2}\frac{\Delta^2}{E^2_{\mathbf{k}}}\Big(\frac{\partial f(E_{\mathbf{k}\uparrow})}{\partial E_{\mathbf{k}\uparrow}}+\frac{\partial f(E_{\mathbf{k}\downarrow})}{\partial E_{\mathbf{k}\downarrow}}\Big)\tau. \quad (17)$$

To prove the relation presented in the main text, we first prove the relation $P=\frac{2}{3}E$ for a polarized unitary Fermi gas in the superfluid phase, where

$$E=\sum_{\mathbf{k}}(\xi_{\mathbf{k}}-E_{\mathbf{k}})+\frac{\Delta^2}{g}+2\sum_{\mathbf{k}}E_{\mathbf{k}}\bar{f}(E_{\mathbf{k}})+\mu n \quad (18)$$

is the energy density. By integration by parts, we obtain the identities $\sum_{\mathbf{k},\sigma}T\ln(1+e^{-\frac{E_{\mathbf{k}\sigma}}{T}})=\frac{2}{3}\sum_{\mathbf{k}}\frac{k^2}{m}\frac{\xi_{\mathbf{k}}}{E_{\mathbf{k}}}\bar{f}(E_{\mathbf{k}})$ and $\sum_{\mathbf{k}}(\xi_{\mathbf{k}}-E_{\mathbf{k}}+\frac{\Delta^2}{2\epsilon_{\mathbf{k}}})=-\frac{1}{3m}\sum_{\mathbf{k}}k^2\left(1-\frac{\xi_{\mathbf{k}}}{E_{\mathbf{k}}}-\frac{\Delta^2}{2\epsilon^2_{\mathbf{k}}}\right)$. At the mean-field level, the pressure P is given by

$$P=-\sum_{\mathbf{k}}(\xi_{\mathbf{k}}-E_{\mathbf{k}})-\frac{\Delta^2}{g}+\sum_{\mathbf{k},\sigma}T\ln(1+e^{-\frac{E_{\mathbf{k}\sigma}}{T}}). \quad (19)$$

Substituting these identities to the expressions of the pressure and energy, and using $\frac{1}{g}=\sum_{\mathbf{k}}\frac{1}{2\epsilon_{\mathbf{k}}}$ in the unitary limit, we obtain

$$E-\frac{3}{2}P=\Delta^2\sum_{\mathbf{k}}\left(\frac{1}{\epsilon_{\mathbf{k}}}-\frac{1}{E_{\mathbf{k}}}+\frac{2}{E_{\mathbf{k}}}\bar{f}(E_{\mathbf{k}})\right)=0, \quad (20)$$

where the gap equation has been applied.

The superfluid density can be obtained from the paramagnetic response function via [11]

$$n_{\mathrm{s}}=m\lim_{\omega\to 0}\lim_{\mathbf{q}\to\mathbf{0}}\mathrm{Re}[\mathcal{P}^{xx}(\omega,\mathbf{q})]+n. \quad (21)$$

For polarized Fermi gases in the BCS-Leggett theory, we found

$$n_{\rm s}=\frac{2\Delta^2}{3m}\sum_{\mathbf{k}}\frac{k^2}{E_{\mathbf{k}}^2}\Big(\frac{1-2\bar{f}(E_{\mathbf{k}})}{2E_{\mathbf{k}}}+\bar{f}'(E_{\mathbf{k}})\Big), \tag{22}$$

where $\bar{f}'(x)=\big(f'(x+h)+f'(x-h)\big)/2$. The shear viscosity characterizes the momentum transfer via the normal density, but the Cooper pairs can also transfer momentum and lead to the anomalous shear viscosity χ [7].

Now we prove the mean-field relation discussed in the main text. Using integration by parts similar to the previous steps, the expression of η becomes

$$\eta=P\tau+\sum_{\mathbf{k}}(\xi_{\mathbf{k}}-E_{\mathbf{k}}+\frac{\Delta^2}{2\epsilon_{\mathbf{k}}})\tau+\frac{1}{15\pi^2 m}\int_0^{+\infty}dk\frac{k^6}{m}\frac{\Delta^2}{E_{\mathbf{k}}^3}\bar{f}(E_{\mathbf{k}})\tau. \tag{23}$$

By applying $E=\frac{3}{2}P$ and integration by parts, we have

$$\begin{aligned}\sum_{\mathbf{k}}(\xi_{\mathbf{k}}-E_{\mathbf{k}}+\frac{\Delta^2}{2\epsilon_{\mathbf{k}}})&=-\frac{2}{5}\Big(-3\sum_{\mathbf{k}}T\ln(1+e^{-\frac{E_{\mathbf{k}}}{T}})+2\sum_{\mathbf{k}}E_{\mathbf{k}}\bar{f}(E_{\mathbf{k}})+\mu n\Big)\\&=-\frac{1}{15\pi^2 m}\int_0^{+\infty}dk\frac{k^6}{m}\frac{\Delta^2}{E_{\mathbf{k}}^3}\bar{f}(E_{\mathbf{k}})-\frac{1}{15\pi^2}\int_0^{+\infty}dk\frac{\Delta^2}{E_{\mathbf{k}}^2}\frac{k^4}{m}\Big[\mu\frac{1-2\bar{f}(E_{\mathbf{k}})}{E_{\mathbf{k}}}-2\xi_{\mathbf{k}}\bar{f}'(E_{\mathbf{k}})\Big].\end{aligned} \tag{24}$$

After substituting this result into Eq. (23), we finally get

$$\begin{aligned}\eta&=P\tau-\frac{2}{15\pi^2}\int_0^{+\infty}dk\frac{\Delta^2}{E_{\mathbf{k}}^2}\frac{k^4}{m}\Big[\mu\frac{1-2\bar{f}(E_{\mathbf{k}})}{2E_{\mathbf{k}}}-\xi_{\mathbf{k}}\bar{f}'(E_{\mathbf{k}})\Big]\tau\\&=P\tau-\frac{2}{5}\mu n_{\rm s}\tau+\frac{2}{15}\sum_{\mathbf{k}}\frac{\Delta^2}{E_{\mathbf{k}}^2}\frac{k^4}{m^2}\bar{f}'(E_{\mathbf{k}})\tau\\&=(P-\frac{2}{5}\mu n_{\rm s})\tau-\chi,\end{aligned} \tag{25}$$

where we have used the mean-field expressions of n_s and χ. Thus, the exact mean-field relation applies to unitary Fermi gases with or without population imbalance.

III. DETAILS OF PAIRING FLUCTUATION THEORY

The MT and AL diagrams for obtaining a gauge-invariant vertex are given as follows.

$$\begin{aligned}\Gamma^\mu_{\rm MT,sc,\sigma}(K+Q,K)&=\sum_L t_{\rm sc}(L)G_{0\bar\sigma}(L-K)\gamma^\mu_{\bar\sigma}(L-K,L-K-Q)G_{0\bar\sigma}(L-K-Q),\\ \Gamma^\mu_{\rm MT,pg,\sigma}(K+Q,K)&=\sum_L t_{\rm pg}(L)G_{0\bar\sigma}(L-K)\gamma^\mu_{\bar\sigma}(L-K,L-K-Q)G_{0\bar\sigma}(L-K-Q),\\ \Gamma^\mu_{\rm AL,1,\sigma}(K+Q,K)&=-\sum_{L,M}t_{\rm pg}(L)t_{\rm pg}(L+Q)G_{0\bar\sigma}(L-K)G_\sigma(L-M)G_{0\bar\sigma}(M+Q)\gamma^\mu_{\bar\sigma}(M+Q,M)G_{0\bar\sigma}(M),\\ \Gamma^\mu_{\rm AL,2,\sigma}(P+Q,P)&=-\sum_{L,M}t_{\rm pg}(L)t_{\rm pg}(L+Q)G_{0\bar\sigma}(L-K)G_{0\bar\sigma}(L-M)G_\sigma(M+Q)\Gamma^\mu_\sigma(M+Q,M)G_\sigma(M),\end{aligned} \tag{26}$$

where $t_{\rm sc}$ and $t_{\rm pg}$ are the t-matrices associated with the condensed and non-condensed pairs, respectively. Moreover, the AL and $\rm MT_{pg}$ diagrams satisfy an identity [12]

$$q_\mu\Big[\frac{1}{2}\Gamma^\mu_{\rm AL,1,\sigma}(K+Q,K)+\frac{1}{2}\Gamma^\mu_{\rm AL,2,\sigma}(K+Q,K)+\Gamma^\mu_{\rm MT,pg,\sigma}(K+Q,K)\Big]=0, \tag{27}$$

which brings further simplifications to our evaluation of the shear viscosity.

Including the pairing fluctuation effects, the expression of the paramagnetic response function is given by

$$\begin{aligned}\overset{\leftrightarrow}{\mathcal{P}}^{ij}(\omega,\mathbf{q})=\sum_{\mathbf{k}}\frac{\mathbf{k}^i\mathbf{k}^j}{2m^2}\Big\{&\Big(1-\frac{\xi^+_{\mathbf{k}}\xi^-_{\mathbf{k}}+\Delta^2_{\rm sc}-\Delta^2_{\rm pg}}{E^+_{\mathbf{k}}E^-_{\mathbf{k}}}\Big)\Big(\frac{1-f(E^+_{\mathbf{k}\uparrow})-f(E^-_{\mathbf{k}\downarrow})}{\omega-E^+_{\mathbf{k}\uparrow}-E^-_{\mathbf{k}\downarrow}}-\frac{1-f(E^+_{\mathbf{k}\downarrow})-f(E^-_{\mathbf{k}\uparrow})}{\omega+E^+_{\mathbf{k}\downarrow}+E^-_{\mathbf{k}\uparrow}}\Big)\\&-\Big(1+\frac{\xi^+_{\mathbf{k}}\xi^-_{\mathbf{k}}+\Delta^2_{\rm sc}-\Delta^2_{\rm pg}}{E^+_{\mathbf{k}}E^-_{\mathbf{k}}}\Big)\Big(\frac{f(E^+_{\mathbf{k}\uparrow})-f(E^-_{\mathbf{k}\uparrow})}{\omega-E^+_{\mathbf{k}\uparrow}+E^-_{\mathbf{k}\uparrow}}-\frac{f(E^+_{\mathbf{k}\downarrow})-f(E^-_{\mathbf{k}\downarrow})}{\omega+E^+_{\mathbf{k}\downarrow}-E^-_{\mathbf{k}\downarrow}}\Big)\Big\}.\end{aligned} \tag{28}$$

In the mean-field BCS-Leggett theory, $\Delta_{\rm pg}=0$ and $\Delta_{\rm sc}=\Delta$, and this expression reduces to Eq. (11).

The shear viscosity acquire two contributions, $\eta=\eta_{\rm f}+\eta_{\rm b}$. The fermionic contribution to the shear viscosity, including the fermionic quasiparticles and condensed pairs, can be evaluated from Eq. (2) in the main text. In the limit $q\to 0$, we have

$$E^{+}_{\mathbf{k}\sigma}-E^{-}_{\mathbf{k}\sigma}=E^{+}_{\mathbf{k}}-E^{-}_{\mathbf{k}}=\mathbf{q}\cdot\nabla E_{\mathbf{k}}=\frac{\mathbf{k}\cdot\mathbf{q}}{m}\frac{\xi_{\mathbf{k}}}{E_{\mathbf{k}}}=\frac{pq\cos\theta}{m}\frac{\xi_{\mathbf{k}}}{E_{\mathbf{k}}}. \tag{29}$$

To derive the expression of the shear viscosity, we need to regularize the δ-function coming from the imaginary part of the current-current response function by

$$\delta(\omega\pm\mathbf{q}\cdot\nabla_{\mathbf{k}}E)=\lim_{\Gamma\to 0}\frac{\frac{1}{\pi}\Gamma}{(\omega\pm\mathbf{q}\cdot\nabla E_{\mathbf{k}})^2+\Gamma^2}. \tag{30}$$

Hence, the shear viscosity becomes

$$\begin{aligned}\eta_{\rm f}=&-m^2\lim_{\omega\to 0}\lim_{q\to 0}\frac{\pi\omega}{2q^2}\sum_{\mathbf{k}}\frac{k^2\sin^2\theta}{m^2}\Big[\frac{E^{+}_{\mathbf{k}}E^{-}_{\mathbf{k}}-\xi^{+}_{\mathbf{k}}\xi^{-}_{\mathbf{k}}-\Delta^2_{\rm sc}+\Delta^2_{\rm pg}}{2E^{+}_{\mathbf{k}}E^{-}_{\mathbf{k}}}\\&\times\big(1-f(E^{+}_{\mathbf{k}\downarrow})-f(E^{-}_{\mathbf{k}\uparrow})\big)\delta(\omega+E^{+}_{\mathbf{k}\downarrow}+E^{-}_{\mathbf{k}\uparrow})-\big(1-f(E^{+}_{\mathbf{k}\uparrow})-f(E^{-}_{\mathbf{k}\downarrow})\big)\delta(\omega-E^{+}_{\mathbf{k}\uparrow}-E^{-}_{\mathbf{k}\downarrow})\big)\\&-\frac{E^{+}_{\mathbf{k}}E^{-}_{\mathbf{k}}+\xi^{+}_{\mathbf{k}}\xi^{-}_{\mathbf{k}}+\Delta^2_{\rm sc}-\Delta^2_{\rm pg}}{2E^{+}_{\mathbf{k}}E^{-}_{\mathbf{k}}}\big(f(E^{+}_{\mathbf{k}\downarrow})-f(E^{-}_{\mathbf{k}\downarrow})\big)\delta(\omega+E^{+}_{\mathbf{k}\downarrow}-E^{-}_{\mathbf{k}\downarrow})-\big(f(E^{+}_{\mathbf{k}\uparrow})-f(E^{-}_{\mathbf{k}\uparrow})\big)\delta(\omega-E^{+}_{\mathbf{k}\uparrow}+E^{-}_{\mathbf{k}\uparrow})\big)\Big]\\=&-\frac{1}{30\pi m^2}\int_0^\infty dkk^6\Big(1-\frac{\Delta^2_{\rm pg}}{E^2_{\mathbf{k}}}\Big)\frac{\xi^2_{\mathbf{k}}}{E^2_{\mathbf{k}}}\lim_{\omega\to 0}\lim_{q\to 0}\big(\frac{\partial f(E_{\mathbf{k}\downarrow})}{\partial E_{\mathbf{k}\downarrow}}\delta(\omega+\mathbf{q}\cdot\nabla E_{\mathbf{k}})+\frac{\partial f(E_{\mathbf{k}\uparrow})}{\partial E_{\mathbf{k}\uparrow}}\delta(\omega-\mathbf{q}\cdot\nabla E_{\mathbf{k}})\big)\\=&\frac{1}{30\pi^2m^2}\int_0^\infty dkk^6\Big(1-\frac{\Delta^2_{\rm pg}}{E^2_{\mathbf{k}}}\Big)\frac{\xi^2_{\mathbf{k}}}{E^2_{\mathbf{k}}}\Big[-\frac{\partial f(E_{\mathbf{k}\uparrow})}{\partial E_{\mathbf{k}\uparrow}}-\frac{\partial f(E_{\mathbf{k}\downarrow})}{\partial E_{\mathbf{k}\downarrow}}\Big]\tau.\end{aligned} \tag{31}$$

Note the δ-functions in the second line do not contribute because $E^{+}_{\mathbf{k}\sigma}+E^{-}_{\mathbf{k}\bar{\sigma}}=E^{+}_{\mathbf{k}}+E^{-}_{\mathbf{k}}>2\Delta$ but $\omega\to 0$, so the arguments do not vanish.

The bosonic contribution is from the noncondensed pairs, and it can be obtained by considering the shear viscosity of a gas of composite bosons with the Hamiltonian $H_{\rm b}=\sum_{\mathbf{q}}\Omega_{\mathbf{q}}b^{\dagger}_{\mathbf{q}}b_{\mathbf{q}}$. Here $b_{\mathbf{q}}$ is the effective annilation operator for the composite bosons. The bosonic Green's function is then given by $G_{\rm b}(i\Omega_l,\mathbf{q})=\frac{1}{i\Omega_l-\Omega_{\mathbf{q}}}$. The current operator is $\mathbf{J}_{\rm b}(\bar{\tau},\mathbf{q})=-\frac{1}{M^*}\sum_{\mathbf{k}}(\mathbf{k}+\frac{\mathbf{q}}{2})b^{\dagger}_{\mathbf{k}}(\bar{\tau})b_{\mathbf{k}+\mathbf{q}}(\bar{\tau})$. This defines a $\mathbf{J}-\mathbf{J}$ linear response, and the response function is given by

$$\overleftrightarrow{Q}^{\mathbf{JJ}}_{\rm b}(\bar{\tau}-\bar{\tau}',\mathbf{q})=-i\theta(\bar{\tau}-\bar{\tau}')\langle[\mathbf{J}_{\rm b}(\bar{\tau},\mathbf{q}),\mathbf{J}_{\rm b}(\bar{\tau}',-\mathbf{q})]\rangle. \tag{32}$$

Finally, the shear viscosity from the noncondensed pairs can be obtained from the transverse part of the $\mathbf{J}-\mathbf{J}$ response function:

$$\eta_{\rm b}=-M^{*2}\lim_{\omega\to 0}\lim_{q\to 0}{\rm Im}\frac{\omega}{q^2}Q^{\mathbf{JJ}}_{\rm bT}(\omega,\mathbf{q})=-\frac{1}{30\pi^2M^{*2}}\int_0^\infty dkk^6\frac{\partial b(\Omega_{\mathbf{k}})}{\partial\Omega_{\mathbf{k}}}\tau. \tag{33}$$

When pairing fluctuations are taken into account, the pressure is decomposed into $P_{\rm f}$ given by Eq. (19) and $P_{\rm b}=-T\sum_{\mathbf{q}}\ln(1-e^{-\frac{\Omega_{\mathbf{q}}}{T}})$ from the pressure of noncondensed pairs using the composite-boson approximation. The anomalous shear viscosity is mainly from the Cooper pairs, so we take the mean-field expression (17) and approximate it by

$$\chi\approx-\frac{1}{15}\sum_{\mathbf{k}}\frac{k^4}{m^2}\frac{\Delta^2_{\rm sc}}{E^2_{\mathbf{k}}}\Big(\frac{\partial f(E_{\mathbf{k}\uparrow})}{\partial E_{\mathbf{k}\uparrow}}+\frac{\partial f(E_{\mathbf{k}\downarrow})}{\partial E_{\mathbf{k}\downarrow}}\Big)\tau. \tag{34}$$

In our approximation, the anomalous shear viscosity χ only includes the contribution from the condensed pairs, and it vanishes above T_c since $\Delta_{\rm sc}(T>T_c)=0$. When $T\to 0$, all the quantities reduce to the mean-field results.

[1] L. P. Kadanoff and P. C. Martin, Annals of Physics **24**, 419 (1963).
[2] Y. He and K. Levin, Phys. Rev. B **89**, 035106 (2014).
[3] H. Guo, Y. Li, Y. He, and C. Chien, J. Phys. B: At. Mol. Opt. Phys. **47**, 085302 (2014).

[4] Y. Nambu, Phys. Rev. **117**, 648 (1960).
[5] J. R. Schrieffer, *Theory of superconductivity* (Benjamin, New York, 1964).
[6] H. Guo, C. C. Chien, and Y. He, J. Low Temp. Phys. **172**, 5 (2013).
[7] H. Guo, W. Cai, Y. He, and C. C. Chien, Phys. Rev. A **95**, 033638 (2017).
[8] L. P. Kadanoff and P. C. Martin, Phys. Rev. **124**, 670 (1961).
[9] K. Levin, Q. J. Chen, C. C. Chien, and Y. He, Ann. Phys. **325**, 233 (2010).
[10] H. Guo, D. Wulin, C. C. Chien, and K. Levin, New J. Phys. **13**, 075011 (2011).
[11] A. L. Fetter and J. D. Walecka, *Quantum Theory of Many-Particle Systems* (Dover Publications, 2003).
[12] I. Kosztin, Q. J. Chen, Y.-J. Kao, and K. Levin, Phys. Rev. B **61**, 11662 (2000).
